# Headache in radiologically isolated syndrome: a hint toward imminent conversion: a case report

**DOI:** 10.1186/s13256-025-05023-6

**Published:** 2025-01-18

**Authors:** Masoud Etemadifar, Hasan Kaveyee, Parto Zohrabi, Amir Mohammad Jozaie, Mehri Salari, Yasin ebne-ali-heydari

**Affiliations:** 1 School of Medicine, Isfahan University of Medical Sciences, Isfahan, Iran; 2 Functional Neurosurgery Research Center, Shohada Tajrish Comprehensive Neurosurgical Center of Excellence, Shahid Beheshti University of Medical Sciences, Tehran, Iran

**Keywords:** Headache, Radiologically isolated syndrome, Multiple sclerosis

## Abstract

**Background:**

Headaches are more prevalent in patients with multiple sclerosis compared with the general population. However, headaches are still considered a rare symptom of multiple sclerosis, especially when they appear as an initial symptom. The occurrence of a headache as a symptom of radiologically isolated syndrome (RIS) is uncommon, and it can significantly increase the likelihood of developing multiple sclerosis.

**Case presentation:**

We report the case of a 36-year-old Iranian woman experiencing severe unilateral headaches without other multiple sclerosis symptoms. Despite normal physical and laboratory exams, cerebrospinal fluid analysis showed positive oligoclonal bands. Magnetic resonance imaging (MRI) revealed multiple demyelinating plaques consistent with RIS. She was treated with dimethyl fumarate. Subsequent MRI confirmed multiple sclerosis by showing new gadolinium-enhanced lesions. After 3 months of dimethyl fumarate treatment, her headache intensity decreased, and she remained otherwise symptom free. Written informed consent was obtained from the patient.

**Conclusion:**

Effectively managing headaches in patients with RIS is a challenge for clinicians to improve their quality of life and delay the progression of multiple sclerosis.

## Introduction

Multiple sclerosis (MS) is a chronic autoimmune neurological disease that mostly affects young adults. Aberrant activation of immune system results in inflammation and demyelination in the central nervous system (CNS), leading to various symptoms of MS [1].

People with multiple sclerosis (pwMS) experience a wide range of symptoms depending on the affected part of the CNS. for instance, headache, optic neuritis (ON), and motor, brainstem–cerebellar, and sensory involvements. Managing these symptoms to enhance the quality of life of pwMS is an important concern for clinicians [2].

While the occurrence of headaches is more common in pwMS compared with the general population, headaches are considered a rare symptom of MS, particularly as an initial symptom [3].

Radiologically isolated syndrome (RIS) is characterized by the presence of white matter lesions in the CNS in the absence of MS clinical symptoms. Individuals with RIS are considered a high-risk population for progression to MS [4].

Herein we aimed to report a complicated case of headache with demyelinating plaques and oligoclonal band (OCB) positive with the absence of other symptoms of MS.

### Case presentation

A 36-year-old Iranian woman was referred to our clinic in February 2024, complaining of severe headaches in the past 4 weeks. Past medical history (PMH) did not include important and specific points. She mentioned the history of long-term headaches in her mother. The patient's headaches were unilateral on left side, radiating from the temporal area to the back of the head. The headaches were non-throbbing and were not accompanied by vomiting and nausea or any other symptoms. Stressful conditions increased their intensity and duration. The headaches did not alleviate after the administration of standard analgesics. The headaches persisted for a duration of 4–5 days and subsequently ceased for an equivalent period. The patient gave a score of 7 out of 10 to the intensity of her headache.

In the physical examination, the fundoscopy was normal, no signs of cranial nerve damage and involvement were seen, and the motor and sensory system did not show any abnormal findings. In the patient’s history, no cause of headache including infection, severe trauma, or serious injury was found. In the laboratory tests, the C-reactive protein (CRP) level was normal, and the sedimentation rate was normal. Angiotensin converting enzyme (ACE) test was negative, and the patient did not have sarcoidosis. Vasculitis test including anti-dsDNA, antinuclear antibody (ANA), anti-phospholipid antibody, anticardiolipin antibody, and beta2glycoprotein antibody were negative. The patient had no signs of infection or lung and other systemic involvement. She experienced no other symptoms except headache.

Her cerebrospinal fluid (CSF) examination also showed positive oligoclonal band (OCB), more than 8 bands compared with serum, and normal levels of glucose and protein CSF/serum ratio. The amount of immunoglobulin G (IgG) was 11/8 g/L (flag) and body fluid IgG index was more than 3/4 (flag). Cytomegalovirus and Epstein–Barr virus antigens were not detected in the CSF.

By magnetic resonance imaging (MRI) examinations, periventricular plaques, tumefactive juxtacortical plaque with large edema, and demyelinated plaques in the cervical region were observed, which show dissemination in space based on MacDonald’s criteria for MS. The result of CSF analysis and MRI were in favor of diagnosis of RIS with high risk for converting to MS. Thus, treatment as a patient with high risk for MS was started with dimethyl fumarate (DMF) 120 mg twice daily for 1 week and after that 240 mg twice daily. The MRI was repeated after a month and this time, several gadolinium enhanced lesions were observed, which showed dissemination in time (DIT) on the basis of McDonald’s criteria. Thus, she was finally diagnosed with MS.

**Fig. 1:**
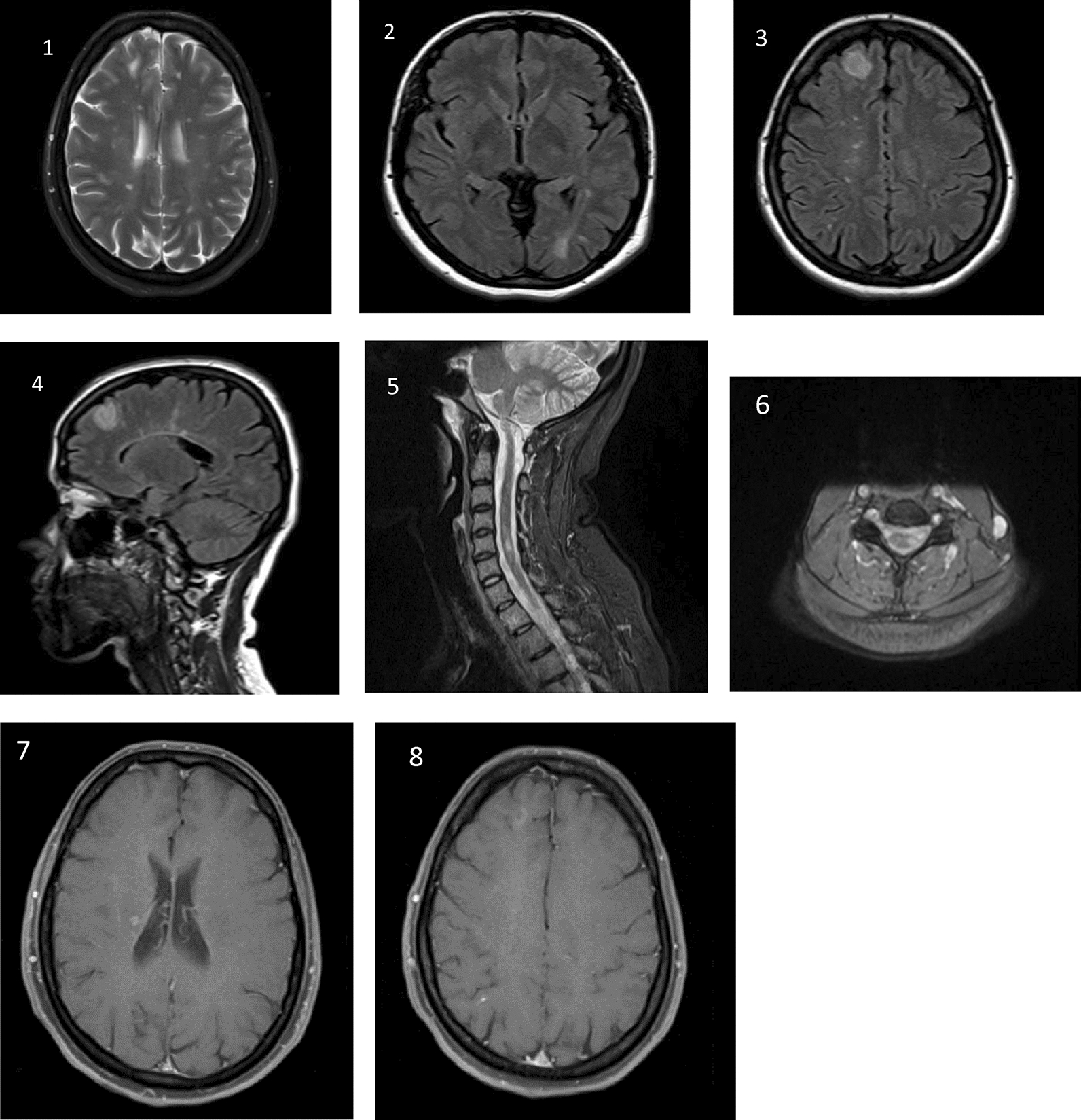
MRI images: axial (1[T2]) and (3[flair]) images showing juxtacortical plaques, axial (2[flair]) showing periventricular plaques, sagittal (4[sagittal]) image showing tumefactive juxtacortical plaques, and (5[stir]) showing cervical plaques. Axial (6) shows spinal plaques, and (7) and (8) show gadolinium-enhanced lesions in brain

After 3 months, Pain intensity decreased from 7 out of 10 to 4 out of 10 and there were no other symptoms or complications in the patient. Written informed consent was obtained from the patient. Figure 1–8 refer to MRI images of the patient.

## Discussion

MS is an immune-mediated disease causing a wide range of symptoms due to CNS involvement. Treatment options are based on controlling disease course with consideration of improving patients’ quality of life and decreasing morbidity [1, 8].

RIS is a condition where MRI scans show abnormalities that indicate demyelination lesions, meeting the criteria for DIS in McDonald’s criteria [5], but without any previous clinical history of demyelinating attacks, ongoing neurological deterioration, or other causes of white matter lesions such as those caused by vascular, infectious, toxic, or drug-related issues [6]. Okuda criteria is used to diagnosis RIS [12].

Several studies have showed that some factors may increase the risk of RIS progressing to MS, for example, factors such as a young age, elevated IgG index, male gender, and involvement of the spinal cord with cervical lesions [7]. It is indicated that the presence of oligoclonal band (OCB) in cerebrospinal fluid (CSF) among patients with RIS may increase their risk of progression to MS within a period of 5 years [8]. A study has revealed that approximately 80% of patients with OCB-positive RIS progress to MS in less than 3 years [5, 6].

The presence of multiple demyelinating plaques in our patient, as observed in RIS, along with the detection of 8 OCB in the CSF, implies an elevated risk of progression to MS. However, except for headache, there were no other symptoms.

Acute MS relapse include symptoms such as optic neuritis (mostly unilateral) in 25% of patients, brainstem symptoms in 45% of them, and partial spinal cord syndromes that are mostly exclusively sensory (for example, sphincter and/or sexual dysfunction) [7]. Further, primary progressive MS often presents with spinal syndrome, a spastic paraparesis, most of the time with no clear sensory level (80–85% cases). Some 10–15% present with progressive cerebellar ataxia, and a smaller number with cognitive, other brainstem, or visual symptoms (2–4%) [8]. The diagnosis may be made only by clinical evidence, here are physical examinations contributing to that:

•Hyperreflexia, extensor plantar responses, lower extremity ataxia, impaired rapid alternating movements, loss of vibration, and proprioception
•Cerebellar tremors of the head or trunk, and cerebellar dysarthria manifested as scanning speech
•Visual loss or afferent pupillary defect secondary to optic neuropathy
•Internuclear ophthalmoplegia, papillitis, and pendular nystagmus
•Intention tremor, spasticity, dysarthria, and paraparesis
•Lhermitte’s sign
•Ascending paresthesia, bandlike tightness

Although headache is not mentioned as an early symptom of MS, some studies have shown a higher prevalence and higher risk of headache in autoimmune diseases including MS, neuromyelitis optica (NMO), and myelin oligodendrocyte glycoprotein (MOG) compared with healthy individuals [8]. Migraine without aura and tension type headache are the most common headaches in MS. Migraine occurs more often in relapsing remitting and tension type tends to occur more often in progressive MS [10].

Potential triggers of headaches in MS include lesions in the pain pathway and mediators of inflammatory processes in the CNS. Furthermore, studies have indicated that the administration of interferon beta (IFN beta), a first-line therapy for MS, can exacerbate headaches in patients with MS [3]. IFN beta use was associated with headache exacerbations in 55% of patients with MS and headache; in another study, this number increased to 73%, so we used DMF for our patient [11, 12]. Younger age shows more risk of headaches duo to IFN beta administration [9]. Thus, using IFN beta in patients with MS and headache should be with care, as it can increase the intensity and duration of headaches.

Studies on headache and RIS are limited. Aguiar et al. [10] reported a case of 28-year-old female patient with new daily persistent headache and RIS. In MRI findings, multiple hyperintense T2 white matter lesions (> 9), including in corpus callosum, juxtacortical, and periventricular (> 3), were noted. None of the lesions were enhanced with gadolinium and patient had no other complications. CSF examination showed 3 OCB bands, which is less than our case. and 2 years later, headaches were persistent and more severe. Despite the use of amitriptyline, topiramate, pregabalin, and gabapentin, patient did not experience improvements. MRI findings showed new T2 white matter lesions enhanced by gadolinium, suggesting the diagnosis of MS. Treatment with IFN beta was started and mild improvement (pain intensity decreased from 5–8 out of 10 to 3–5) was achieved. After 2 years of IFN beta use, patient did not have additional attacks and MRI activity, but headaches persisted during the time, resulting in morbidity. Thus, controlling headache in patients with RIS and MS is an important area of challenge [10].

It is important for clinicians to recognize that not all headaches should be dismissed as simple complaints. Headaches can be an early symptom of RIS, and along with OCB positivity, increase the risk of progressing to MS. Therefore, it is crucial for neurologists to pay special attention to these patients and conduct MRI and CSF tests. Treatment may be initiated on the basis of the patient’s condition and the severity of CSF and MRI findings. However, there is a lack of comprehensive studies in this specific area, and further research is needed to determine the best management strategies for achieving headache relief and preventing the progression to MS.

## Conclusion

Headaches may present as an initial symptom in RIS, increasing the risk of conversion to MS. It emphasizes the importance of diagnostic evaluation for timely diagnosis and appropriate management. Additionally, it is essential for individuals presenting with atypical headaches, especially in the absence of other symptoms of MS or RIS, to undergo specific evaluations for signs of these diseases.

## Data Availability

Data and other materials can be made available if required.
